# Research on the function of GPX4 in tumor-targeted treatment based on its molecular structure and features

**DOI:** 10.3389/fonc.2025.1594234

**Published:** 2025-09-03

**Authors:** He Geng, Lin Ma, Lixin Wu, Cuicui Yao, Can Wang, Xinyan Gan, Yiyi Li, Fang Chen

**Affiliations:** Department of Anaesthesiology, Shenzhen Children’s Hospital, Shenzhen, China

**Keywords:** GPx4, ferroptosis, lung cancer, esophageal cancer, gastric cancer

## Abstract

GPX4 is the only antioxidant enzyme in mammals that can convert cholesterol and phospholipid hydroperoxide into phosphatidylol and cholesterol. Its peculiar molecular structure allows it to perform a variety of biological functions, including oxidative stress, ferroptosis regulation, brain development stimulation, and immune responses. In recent years, several studies have demonstrated that increased GPX4 expression is linked to tumor cell proliferation, growth, migration, and differentiation. Overexpression of GPX4 has been linked to chemoresistance and a poor prognosis in cancers including nasopharyngeal carcinoma, breast cancer, and lung cancer. GPX4 inhibitors have been demonstrated to be effective in the treatment of numerous drug-resistant malignancies. Some tumors, such as glioblastoma, do not react well to single-agent GPX4 inhibitors. Therefore, future research should focus on identifying cancers that are more responsive to GPX4 inhibitors and enhancing the efficacy of these inhibitors. This research investigates the regulatory mechanisms and properties of GPX4 in various tumor cell types, as well as its molecular structure and biological roles, hoping to propose more effective tumor-targeted therapy alternatives.

## Highlights

- GPX4 is a key target for tumor cell multiplication, differentiation, migration, and invasion.
- The GPX4 tumor regulating mechanism is dependent on the modulation of ferroptosis.
- The high level of GPX4 expression is linked to the development of chemical resistance in a range of malignancies as well as the prognosis of tumor patients.

## 1 Introduction

GPX4 is a tetrameric selenoprotein and the key peroxidase enzyme in mammals, converting hazardous lipid peroxides into non-toxic lipid alcohols. GPX4 is necessary for an extensive spectrum of bodily processes, including oxidative stress, ferroptosis regulation, brain development stimulation, and immune responses. The regulation of ferroptosis is an important biological function of GPX4. Dixon et al. (2012) (1) proposed ferroptosis as a novel cell death modality, with the main mechanisms being lipid peroxidation, polyunsaturated fatty acid accumulation, iron overload caused by abnormal cellular metabolism, glutathione depletion, and decreased glutathione peroxidase (GPX4) activity. Cancer is a major threat to human life and health all over the world, and studies have shown that the mechanism of inhibition of tumor proliferation by a variety of chemotherapy adjuvant drugs is closely related to ferroptosis, and further studies have shown that the GPX4 protein, an important regulator of ferroptosis (2–4), plays an important role in the process of tumor growth, proliferation, migration, and differentiation. Targeting GPX4 inhibitors may be a successful treatment strategy for drug-resistant tumors in the future. Furugaki et al. (5) discovered that GPX4 is involved in promoting the survival of DTP cell subsets (tumor drug-resistant cell subsets), indicating that the high expression of GPX4 in tumor cells may be related to the drug resistance of tumor cells. Thus, it’s critical to comprehend GPX4’s molecular makeup, role, and regulatory method on tumor cells. This essay largely begins with the above aspects, in order to bring new ideas and views for tumor targeted therapy.

## 2 GPX4 molecular structure

GPX4, a glutathione peroxidase, is a tetrameric selenoprotein consisting of 170 amino acids and having a molecular weight of approximately 19 kDa. It is the only antioxidant enzyme in mammals that can currently convert cholesterol and phospholipid hydroperoxide into phosphatidylol and cholesterol (6, 7), effectively inhibiting cell ferroptosis and maintaining cell membrane stability. The GPX4 gene is located on human chromosome 19 (19P13.3), with a total gene span of 2.8 kb and seven exons (8). In humans, GPX4 has three isoforms (cGPX4, mGPX4, and snGPX4) found in the cytoplasm, mitochondria, and sperm nucleus, respectively (9).According to Ingold et al. (10), the active site of GPX4 is selenocysteine, and its physiological role is dependent on selenium. They studied the survival times of mice with and without selenocysteine GPX4, and discovered that while the latter could grow and develop properly, it usually died before weaning. Their findings reveal that selenocysteine GPX4, a particular selenoprotein in mammals whose function cannot be substituted by other oxidoreductases, is required for certain brain development after birth. In mammals, GPX4 protein production is slow and energy-intensive. As shown by Zhang et al (11), through the Rag-mTORC4-1EBP signaling axis, SLC7A11-mediated reabsorption of cystine and selenocysteine can activate rapamycin complex 1 (mTORC1), which in turn synthesizes glutathione (GSH) and GPX4 proteins. In brief, GPX4 is the only tetrameric selenium protein in mammals capable of converting harmful lipid peroxides into non-toxic lipid alcohols, crucial for maintaining cell membrane stability and neural development.

## 3 GPX4 features

### 3.1 Oxidative stress and GPX4

**Figure 1:**
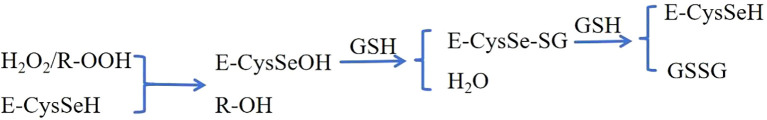
The degradation of peroxide by GPX4.

GPX4 is a membrane-associated phospholipid peroxidase in the glutathione peroxidase (GPX) family. Ursini et al. (12) purified a protein (GPX4) from pig liver and discovered that it was distinct from previous glutathione peroxidase enzymes in that it could inhibit the lipid peroxidation process of mitochondria and microsomes in rat liver and stabilize cell membranes by converting toxic lipid peroxides (R-OOH) into non-toxic lipid alcohols. (R-OH). According to Sattler and Noguchi’s and other academics’ study, the degradation of peroxide by (13, 14) GPX4 mostly includes the following chemical pathways, with selenocysteine (E-CysSeH, selenol) as the reaction center site(**Figure 1**): 1) Hydrogen peroxide or organic hydroperoxide (R-OOH) reacts with selenol (E-CysSeH) to form selenic acid (E-CysSeOH) and corresponding alcohol (R-OH); 2) E-CysSeOH then reacts with GSH to form selenide sulfide (E-CysSe-SG) and H2O; 3) E-CysSe- (GSSG). GPX4’s antioxidant activity is critical for maintaining normal physiological function of cells and the organism.

### 3.2 Ferroptosis and GPX4

**Figure 2:**
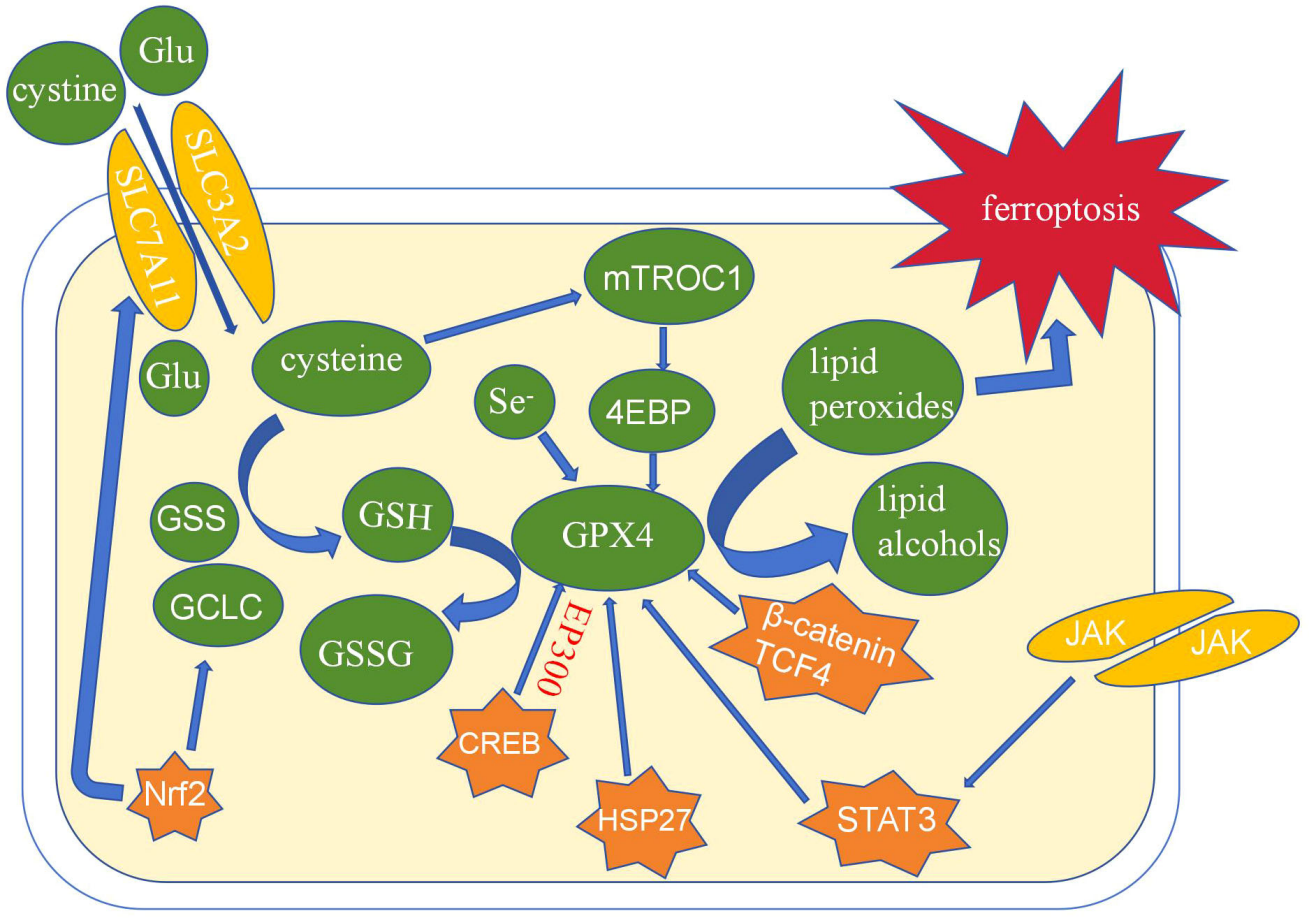
The role of GPX4 in ferroptosis.

Ferroptosis is a type of non-apoptotic cell death characterized by membrane integrity loss, cytoplasmic swelling, increased mitochondrial membrane density, and a diminished or nonexistent mitochondrial crest (1). The investigation of its mechanism is a hot topic in the disease’s present development. GPX4 inhibition is thought to be the primary node for producing ferroptosis among the numerous mechanisms of ferroptosis (15). The Nrf2/System xc-axis is primarily responsible for GPX4’s regulation of ferroptosis. System xc- is a key antioxidant system composed of glycosylated heavy chain SLC3A2 protein and non-glycosylated SLC7A11 protein, which is a glutamate and cystine transporter in the body (16). Cystine delivered into cells by System xc- quickly degrades to cysteine, which is engaged in the production of glutathione (GSH), the regulatory substrate for GPX4. GPX4 eliminates lipid peroxides with the help of glutathione to prevent ferroptosis. By encouraging the production of GPX4, a number of biological variables in organisms, including CREB, HSP27, STAT3, and others, can influence ferroptosis. These elements are directly related to the prognosis and chemical resistance of tumor cells(**Figure 2**). GPX4’s modulation of ferroptosis offers new avenues for cancer treatment.

### 3.3 Neurons and GPX4

GPX4 is crucial for mammalian embryonic neuronal development and adult neuronal health. According to Yant and Imai et al. (2, 17), the GPX4 protein can protect mammals from radiation and oxidative damage during the embryonic stage, and that without the GPX4 protein, more embryos die in the second trimester. Ingold et al. (10) associated early death in GPX4-deficient mice with neuronal dysplasia. Chen et al. (18) confirmed GPX4’s role in maintaining adult mouse nervous system health. They used Tamoxifen (TAM) to remove the GPX4 protein in adult mice. The mice had severe muscle atrophy and fast paralysis after 8 days of TAM therapy, and histological investigation revealed severe degeneration of their spinal motor neurons but no significant changes in neurons in the cerebral cortex. The GPX4 regulation mechanism on neuronal development is closely linked to ferroptosis and contributes to neurological conditions such as seizures, cerebral ischemia-reperfusion injury, and Alzheimer’s disease.

### 3.4 Immune response and GPX4

**Figure 3:**
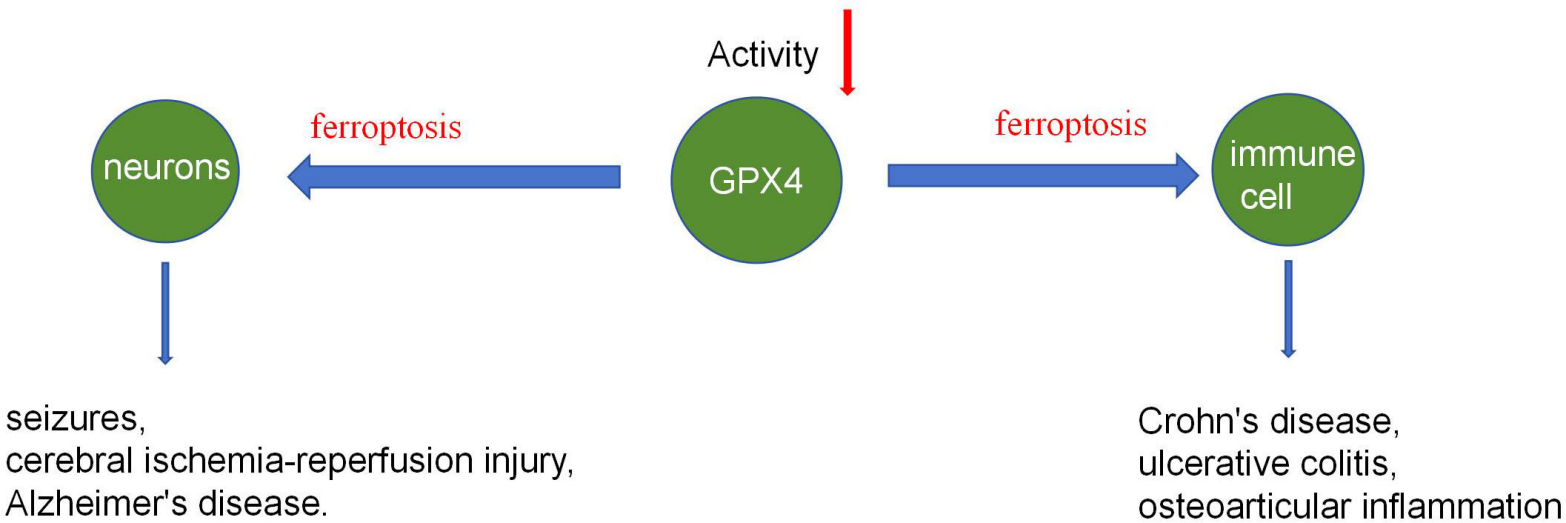
GPX4 regulates neurons and immune cells.

GPX4 is involved in the body’s immune response through various pathways, with GPX4 protein deficiency leading to immune degradation and inflammation. Schwärzler et al. (19) discovered that GPX4 deficiency in mouse fat cells causes spontaneous lipid peroxidation and the expression of inflammatory factors, including TNF-α, IL-1, IL-6, and CXCL1. Mice lacking GPX4 protein showed mild inflammation compared to wild-type mice. Schwärzler’s research found that GPX4 in fat cells improves metabolic disorders and prevents inflammation, unrelated to ferroptosis. STING (interferon gene stimulating factor) is required for the detection of cytoplasmic DNA and the induction of innate immune responses against microbial infections and tumors. Jia et al (20) discovered that GPX4 promotes STING activation by maintaining lipid redox homeostasis. GPX4 protein deficiency inhibits the cGAS-STING pathway and HSV-1-induced antiviral immune responses, leading to HSV-1 replication. Furthermore, GPX4 can promote autoimmunity via controlling neutrophil ferroptosis (21) and boost cellular immunity by suppressing ferroptosis in CD4 T+ cells (22, 23). The control of the immune response by GPX4 is also involved in the etiology of disorders such as Crohn’s disease, ulcerative colitis, and osteoarticular inflammation (24–26) (**Figure 3**).

## 4 The development and progression of GPX4 inhibitors

In 2019, Miotto, Hassannia,et al noted that ferroptosis inhibitors (FINs) fall into four general groups. Class I FINs, like erastin, act by depleting GSH, Class II and Class III FINs are defined as inhibiting GPX4 activity either by directly inactivating the enzyme or by lowering its expression, respectively, while Class IV FINs promote iron overload. Small molecules that trigger ferroptosis include RSL3, ML162, and ML210; these compounds are often used in studies of ferroptosis and are generally considered as GPX4 inhibitors (27, 28).Different protein molecules inhibit GPX4 in different ways, including: RSL3 is thought to be a direct inhibitor of GPX4. By catalyzing the K6-linked polyubiquitination of GPX4, BRCA1 and Timosaponin AIII accelerate the degradation of GPX4 and thereby induce ferroptosis (29, 30). Baicalein inhibits GPX4 expression via the JAK2/STAT3/GPX4 axis (31). By blocking the Janus kinase 2 (JAK2)/STAT3 signaling pathway through direct interaction with JAK2, baicalein (7.5-30μM) dose-dependently reduced the expression levels of GPX4, a key regulator of ferroptosis, in HCT116 and DLD1 cells, ultimately causing ferroptosis in CRC cells, according to Lai, Zhao, et al. The administration of baicalein (10, 20 mg/kg, i.e., every two days for two weeks) significantly decreased the formation of tumors in a CRC xenograft mice model by blocking the JAK2/STAT3/GPX4 axis in tumor tissue.

## 5 GPX4 controls tumor cell growth

The regulation of GPX4 on various types of tumor cells is primarily determined by its regulation of ferroptosis; however, the treatment effect of a single GPX4 inhibitor on some types, such as glioblastoma, is poor, which indicates that the GPX4 protein is not the only target in some types of tumors, and that multiple molecular channels are involved in the growth and proliferation of these tumors. **Table 1** summarizes the impact of GPX4 on each type of tumor.

**Table 1:** The impact of GPX4 on each type of tumor.

Type of tumor	GPX4 expression level	Key regulatory pathways/mechanisms	Drug resistance and prognosis	Sensitive inhibitors
Nasopharyngeal carcinoma	High expression	p62-Keap1-NRF2	drug resistance poor prognosis	/
Breast Cancer	High expression	induce GPX4 ubiquitination	/	TNBC
Lung Cancer	High expression	CREB/EP300/GPX4 induce GPX4 ubiquitination	/	Bufotalin (BT)
Esophageal cancer	High expression	HSP27-GPX4	drug resistance poor prognosis	/
Gastric cancer	High expression	β-catenin/TCF4/GPX4	drug resistance poor prognosis	snake fibulin B (OP-B)
Colorectal Cancer	High expression	IF20A/NUAK1/Nrf2/GPX4	drug resistance poor prognosis	/
Liver Cancer	High expression	lncRNA PVT1/miR-214-3p/GPX4 suppressing System xc-	/	Ketamine,TGF-β1
Pancreatic Cancer	High expression	STAT3-GPX4	/	TST
Kidney Cancer	High expression	/	drug resistance poor prognosis	KLF2
Bladder cancer	High expression	/	/	FIN56
Glioma	High expression	GPX4 degradation via the lysosomal pathway	/	Temozolomide (TMZ) ,Plum protein (PLB)
Glioblastoma	High expression	NF-κB pathway	drug resistance poor prognosis	
Neuroblastoma	High expression	High MYCN expression	/	/
Osteosarcoma	High expression	HMOX1-GPX4	/	EF24

### 5.1 Nasopharyngeal carcinoma and GPX4

Nasopharyngeal carcinoma is sensitive to chemoradiotherapy, with a cure rate of about 70%, and Yang et al. (32) discovered that about 30% of patients with advanced nasopharyngeal carcinoma will develop chemotherapy resistance. Patients with advanced distant migration have a poor prognosis, and their treatment options are critical issues that must be resolved as soon as possible. Yuan et al. (33) discovered that *in vivo* redox homeostasis has a significant impact on nasopharyngeal carcinoma caused by EBV infection, and that GPX4 deficiency can inhibit nasopharyngeal cancer cell proliferation. EBV can increase the expression of SLC7A11 and GPX4 by activating the p62-Keap1-NRF2 signaling pathway, making nasopharyngeal cancer cells more resistant to ferroptosis. This implies that GPX4 is associated with chemoresistance in nasopharyngeal carcinoma cells.

### 5.2 Breast cancer and GPX4

Breast cancer is the most common cancer in women and the leading cause of cancer death. TNBC is the most aggressive type with high recurrence and mortality rates (34), and chemotherapy is the backbone of treatment (35). Ding et al. (36) discovered that by directly binding to GPX4, derivatives of the natural substance feverflowylide (DMOCPTL) can induce GPX4 ubiquitination, lower GPX4 expression levels, and accelerate ferroptosis in breast cancer cells. They evaluated the levels of GPX4 protein expression in breast cancer cells and discovered that TNBC cells showed higher GPX4 expression than non-TNBC cells, with GPX4 deficiency inducing ferroptosis. Ultimately, the GPX4 protein is a novel target for breast cancer therapy, particularly TNBC, and it offers a novel strategy for TNBC treatment by reducing GPX4 expression and inducing ferroptosis.

### 5.3 Lung cancer and GPX4

Lung cancer is the leading cause of cancer-related deaths globally, especially in males. About 11% of patients are diagnosed with a set of histologic subtypes known as non-small cell lung cancer (NSCLC), with lung adenocarcinoma (LUAD) being the most prevalent subtype (37). Wang et al. (38) demonstrated the role of the CREB/EP300/GPX4 axis in the therapy of lung cancer using western blotting (IB), immunohistochemistry (IHC), and enzyme-linked immunosorbent assay (ELISA). CREB is a transcription factor that is found all over the body. CREB has a positive regulatory effect on GPX4 and promotes GPX4 transcription by binding to the GPX4 promoter, which is aided by EP300. This suggests that targeting this axis may offer new treatment options for LUAD. Zhang et al. (39) discovered a link between GPX4 and non-small cell lung cancer. Bufotalin (BT), a naturally occurring small molecule, has been shown in studies to induce ferroptosis in non-small cell lung cancer, with the mechanism relying on BT-induced GPX4 ubiquitination. By accelerating the degradation of GPX4, increasing intracellular Fe, and causing lipid peroxidation, bofantaline can induce ferroptosis in cancer cells. BT’s ability to cause lipid peroxidation was significantly reduced in GPX4 deficiency, indicating that BT is dependent on GPX4 for inducing ferroptosis in non-small cell lung cancer. In short, GPX4 is linked to the occurrence and progression of lung cancer and may become a new target for lung cancer targeted therapy.

### 5.4 Esophageal cancer and GPX4

Esophageal carcinoma is one of the most aggressive gastrointestinal malignancies. Despite surgical resection and adjuvant therapy, disease recurrence is common, five-year overall survival is low, and the prognosis is bleak (40). The high aggressiveness and activity of esophageal cancer cells is linked to the cancer stem cell theory, which states that a subset of cancer cell subsets possess stem cell traits. These cancer stem cells cause tumors to be resistant to chemoradiotherapy and to be recurring and aggressive. Liu et al. (41) demonstrated that esophageal cancer cells in spheroid culture medium had cancer stem cell features, which is also the fundamental reason why esophageal cancer is difficult to cure. Another study (42) found that stem cell features in esophageal cancer are linked to HSP27-GPX4 pathway-mediated ferrosis. Esophageal cancer stem cells displayed strong lipid peroxidation, elevated iron concentration, and high GPX4 expression. The highly expressed GPX4 protein suppressed ferroptosis in esophageal cancer stem cells, suggesting chemoradiotherapy resistance. Simultaneously, Liu et al. noted that GPX4 has some usefulness in predicting patient prognosis, and patients with high GPX4 expression have a worse prognosis. As a result, targeting GPX4 expression may be one of the treatment methods for substantially curing esophageal cancer and improving patients’ prognoses.

### 5.5 Gastric cancer and GPX4

Gastric cancer is the fourth leading cause of death from cancer worldwide (43). In Asia, cisplatin-based chemotherapy is the first-line treatment option for advanced gastric cancer (44), but resistance to chemotherapy (45) has always been a problem in clinical treatment. By regulating ferroptosis, GPX4 can prevent the development of gastric cancer and chemical resistance. Zhang et al. (46) discovered that snake fibulin B (OP-B), a substance extracted from Japanese red beans, can induce ferroptosis in gastric cancer cells by inhibiting GPX4 expression. Wang et al. (47) discovered the role of the β-catenin/TCF4/GPX4 axis in gastric cancer chemoresistance. By binding to GPX4 promoters,β-catenin and TCF4 transcription factor complexes can promote GPX4 expression, inhibiting ferroptosis and making cancer cells resistant to drugs. At the same time, high GPX4 expression was associated with a poor prognosis in gastric cancer patients. Sugezawa et al. (48) used tumor samples from gastric cancer patients to confirm that high expression of GPX4 is an independent risk factor for postoperative survival. In conclusion, the GPX4 protein is linked to the occurrence and progression of gastric cancer, as well as gastric cancer chemodrug resistance and patient prognosis, and targeted GPX4 blockade improves gastric cancer chemosensitivity and overall survival rate.

### 5.6 Colorectal cancer and GPX4

Colorectal cancer is the second leading cause of cancer death worldwide. Currently, the guidelines recommend surgical resection as the mainstay of treatment for patients with advanced colorectal cancer, supplemented by chemotherapy. The first-line chemotherapy regimen is based on oxaliplatin. However, due to colorectal cancer’s chemoresistance, only 40% of patients benefit from it (49).Yang et al. (50) discovered that GPX4 is associated with chemoresistance in colorectal cancer, and that inhibiting the IF20A/NUAK1/Nrf2/GPX4 signaling pathway can increase cancer cell sensitivity to oxaliplatin. GPX4 expression inhibition can induce ferroptosis in cancer cells and inhibit colorectal cancer proliferation (51). The regulation of GPX4 in colorectal cancer is mostly dependent on ferroptosis. Targeting GPX4 to induce ferroptosis could inhibit colorectal cancer proliferation and overcome chemoresistance.

### 5.7 Liver cancer and GPX4

Liver cancer is one of the top four malignant malignancies worldwide. Surgery, radiation therapy, and chemotherapy are all options for treatment. He et al. (52) analyzed tumor cell samples with non-tumor cell samples from liver cancer patients and discovered that GPX4 protein displayed high expression levels in liver tumor cells and that ketamine can induce hemozois in hepatocellular cancer cells via the lncRNA PVT1/miR-214-3p/GPX4 axis. Conche et al. (53) discovered that GPX4 deletion induces ferroptosis and tumor-suppressive immune responses and that the two operate synergistically to decrease hepatoma cell proliferation. The regulation of GPX4 on liver cancer is mostly dependent on ferroptosis, and targeted suppression of GPX4 may be a novel path for non-surgical liver cancer treatment. TGF-β1 (transforming growth factor β1) can increase hepatoma cell sensitivity to GPX4 inhibitors by suppressing System xc-, which will benefit GPX4-targeted therapy for hepatoma cells (54).

### 5.8 Pancreatic cancer and GPX4

Pancreatic cancer is a malignant tumor that begins slowly, grows quickly, and is highly aggressive, rendering surgery unsuitable for the majority of pancreatic cancer patients. Pancreatitis is a known risk factor for pancreatic cancer (55). Zhang et al. (56) discovered that TST (an antibiotic derived from Streptomyces) can reduce pancreatic cancer cell proliferation by inhibiting the STAT3-GPX4 signaling pathway. STAT3 is one of the molecules that regulates GPX4. TST strongly decreases STAT3 protein expression and phosphorylation, limiting GPX4 transcription and enhancing ferroptosis in pancreatic cancer cells, according to western blot tests. By using N6F11 as a degrader of GPX4 in cancer cells, the study by Li et al. (57) also confirmed that targeted degradation of GPX4 can be used to treat pancreatic cancer. This degrader causes ferroptosis in cancer cells, but it does not degrade GPX4 in immune cells, allowing the body to maintain its immune response to tumors. In summary, GPX4 may play a significant role in the development and occurrence of pancreatic cancer. Excessive GPX4 protein consumption causes pancreatitis, which in turn encourages Kras-driven pancreatic carcinogenesis. The predictive survival rate of patients with pancreatic cancer can be significantly increased by the high expression of GPX4 in these cells.

### 5.9 Kidney cancer and GPX4

Kidney cancer, particularly renal clear cell carcinoma, is common in the urinary system. Su et al. (58) discovered that, as compared to normal kidney cells, GPX4 is overexpressed in renal cancer cells and promotes their proliferation and metastasis. KLF2 is a tumor suppressor gene that reduces the growth, migration, and invasion of renal clear cell carcinoma.KLF2 and GPX4 have been linked to the migration and invasion of renal clear cell carcinoma, according to Lu et al. (59). KLF2 and GPX4 expression have been found to be negatively correlated, which can cause ferroptosis in renal clear cell carcinoma by regulating GPX4 transcription. *In vivo*, KFL2 overexpression significantly inhibits GPX4 transcription, preventing cancer cells from migrating and invading. The regulation of renal cancer cells by GPX4 is primarily dependent on ferroptosis, and high GPX4 expression may be associated with renal clear cell carcinoma proliferation and metastasis. Inhibiting GPX4 transcription can be used as a novel target for treating kidney cancer and reducing cancer migration and invasion.

### 5.10 Bladder cancer and GPX4

Bladder cancer is the world’s tenth most frequent cancer, with an estimated 549,000 new cases and 200,000 deaths per year. FIN56 is a ferroptosis inducer that has been linked to GPX4 degradation via ferroptosis-inducing processes (60). Further research by Sun et al. revealed that (61) FIN56 can stimulate GPX4 degradation, increasing ferroptosis in bladder cancer cells, implying that GPX4 can be exploited as a therapeutic target for bladder cancer medicines. However, the mechanism through which FIN56 causes GPX4 degradation remains unknown. There are currently no studies that link GPX4 to bladder cancer growth, invasion, or prognosis. Based on the link between GPX4 and other malignancies, GPX4 inhibitors may be extremely useful in slowing the progression of bladder cancer and improving prognosis.

### 5.11 Glioma and GPX4

Glioma, the most common malignant brain tumor, has poor prognosis. Adjuvant chemoradiotherapy, in addition to surgical resection, is currently used to treat glioma. Temozolomide (TMZ) is the first-line treatment for glioma.Hu et al. (62) demonstrated that glioma resistance to temozolomide is associated to ferroptosis, implying that inducing glioma ferroptosis is critical for clinical therapy and drug resistance. Plum protein (PLB) has been shown to decrease tumor growth and proliferation (63, 64).PLB, according to Zhan et al. (65), can inhibit glioma growth *in vitro* via the ferroptosis pathway, and its mechanism of producing ferroptosis is dependent on GPX4 degradation via the lysosomal pathway. This shows that GPX4 could be employed as a therapeutic target for glioma.

### 5.12 Glioblastoma and GPX4

Glioblastoma is the most common primary nervous system cancer. It is extremely aggressive, has a high rate of recurrence, and is currently difficult to treat. RAS-selective lethal 3 (RSL3), a well-known inhibitor of glutathione peroxidase 4 (GPX4), could effectively induce oxidative cell death in glioblastoma cells through ferroptosis. Li et al. (66) found that NF-κB pathway plays a novel role in RSL3-induced ferroptosis in glioblastoma cells. Additionally, their studies indicated that glioblastoma ferroptosis could not be efficiently induced by only knocking down the GPX4 gene. Ferroptosis happens in glioblastoma when the NF-κB pathway is triggered while the GPX4 gene is silenced. This could be the cause of the poor survival and chemoresistant nature of glioblastoma.

### 5.13 Neuroblastoma and GPX4

Neuroblastoma is the most frequent extracranial malignancy in neonates, with 50% of patients experiencing tumor recurrence. Despite the availability of adjuvant multimodality therapy, overall survival is only 40% (67).In neuroblastoma, the oncogene MYCN is extensively expressed. Neuroblastoma cells with high MYCN gene expression were reported to be sensitive to GPX4-targeted ferroptosis inducers, according to Lu et al. (68). GPX4 protein could be a good target for neuroblastoma drugs. At the same time, the MYCN gene is strongly linked to patient prognosis, and increased MYCN expression leads to tumor recurrence and a bad prognosis. The two might work in concert to treat neuroblastoma, which has a better prognosis than glioblastoma and is more responsive to chemotherapy. A better knowledge of the association between the GPX4 protein and neuroblastoma will assist solve medication resistance, high recurrence, and enhance neuroblastoma prognosis.

### 5.14 Osteosarcoma and GPX4

Osteosarcoma is the most common primary bone cancer that arises from bone stromal cells and is more common in people under the age of 20. Osteosarcoma has a five-year survival rate of 68 percent, a decline of about 1.3 percent per year, and it accounts for 8.9 percent of all cancer fatalities in children and adolescents (69).EF24 is a curcumin analogue with apoptotic capabilities, as well as the ability to prevent cancer proliferation and migration. Lin et al. discovered that (70) EF24 can block GPX4 expression by upregulating HMOX1, causing osteosarcoma cells to undergo ferrosis. This demonstrates that the GPX4 protein can be utilized as a target for osteosarcoma medicines. However, there have been few research on GPX4 protein inhibitors and osteosarcoma, therefore it can only be postulated that GPX4 may be involved in the prevention of osteosarcoma cell proliferation, migration, and invasion by acting as the key node for triggering ferroptosis. More trials are needed to determine whether GPX4 inhibitors can benefit osteosarcoma patients.

## 6 Summary and prospects

As the sole redidoreductase in the body capable of converting hazardous lipid peroxides into non-toxic lipid alcohols, GPX4 plays an important role in a range of biological activities, particularly regulating redox homeostasis and cell membrane stability. The regulation of ferroptosis is an important biological function of GPX4 (71). In tumor therapy, GPX4 is a crucial targeted molecule. Its control over ferroptosis is mostly responsible for its control over malignancies. Cellular ferroptosis results from lipid peroxide buildup in cells caused by GPX4 deficiency. Numerous tumor cells, including esophageal, lung, and nasopharyngeal cancers, have significant expression of the GPX4 factor. Utilizing this, targeted reduction of GPX4 expression in tumor cells can be regarded as a crucial tumor therapeutic approach. Experiments showed that activation of the NF-κB pathway also plays a significant role in the incidence of glioblastoma ferroptosis, and that merely knocking out the GPX4 gene was insufficient to cause effective ferroptosis in cancer cells in some tumors, such as glioblastoma. Furthermore, GPX4’s control of ferroptosis in tumor cells is also significantly impacted by alterations in the microenvironment within those tumor cells, including variations in lipid peroxide and iron ion levels. These elements might be crucial for the development of chemical resistance in malignancies and a bad prognosis. One of the key issues in modern cancer treatment is resolving tumor medication resistance and enhancing the prognosis of cancer patients. The effects of GPX4 inhibitors on tumors have been the primary focus of previous research. However, there is a therapeutic difference between GPX4 inhibitors and other tumor treatments. For instance, tumor cells are more susceptible to GPX4 inhibitors in the therapy of neuroblastoma because of the high expression of the tumor’s own MYCN gene. In a similar vein, Gu et al.’s study revealed that GPX4 and PARP inhibitors worked in concert, increasing DNA damage and ultimately killing cancer cells with functional HR pathways by a significant margin (72). Additionally, GPX4 and CDK4/6 inhibitors work in concert to treat breast cancer (73). Thus, more research can look at the potential for tumor-targeted treatments, including how GPX4 and other cell death mechanisms are regulated, as well as how GPX4 inhibitors and immune checkpoint inhibitors work in concert.
